# An Evolutionarily Conserved Sexual Signature in the Primate Brain

**DOI:** 10.1371/journal.pgen.1000100

**Published:** 2008-06-20

**Authors:** Björn Reinius, Peter Saetre, Jennifer A. Leonard, Ran Blekhman, Roxana Merino-Martinez, Yoav Gilad, Elena Jazin

**Affiliations:** 1 Department of Development and Genetics, Uppsala University, Uppsala, Sweden; 2 Department of Clinical Neuroscience, HUBIN Project, Karolinska Institutet and Hospital, Stockholm, Sweden; 3 Department of Evolutionary Biology, Uppsala University, Uppsala, Sweden; 4 Department of Human Genetics, University of Chicago, Chicago, Illinois, United States of America; 5 Department of Molecular Medicine and Surgery, Karolinska Institutet and Hospital, Stockholm, Sweden; The University of Queensland, Australia

## Abstract

The question of a potential biological sexual signature in the human brain is a heavily disputed subject. In order to provide further insight into this issue, we used an evolutionary approach to identify genes with sex differences in brain expression level among primates. We reasoned that expression patterns important to uphold key male and female characteristics may be conserved during evolution. We selected cortex for our studies because this specific brain region is responsible for many higher behavioral functions. We compared gene expression profiles in the occipital cortex of male and female humans (*Homo sapiens*, a great ape) and cynomolgus macaques (*Macaca fascicularis*, an old world monkey), two catarrhine species that show abundant morphological sexual dimorphism, as well as in common marmosets (*Callithrix Jacchus*, a new world monkey) which are relatively sexually monomorphic. We identified hundreds of genes with sex-biased expression patterns in humans and macaques, while fewer than ten were differentially expressed between the sexes in marmosets. In primates, a general rule is that many of the morphological and behavioral sexual dimorphisms seen in polygamous species, such as macaques, are typically less pronounced in monogamous species such as the marmosets. Our observations suggest that this correlation may also be reflected in the extent of sex-biased gene expression in the brain. We identified 85 genes with common sex-biased expression, in both human and macaque and 2 genes, X inactivation-specific transcript (XIST) and Heat shock factor binding protein 1 (HSBP1), that were consistently sex-biased in the female direction in human, macaque, and marmoset. These observations imply a conserved signature of sexual gene expression dimorphism in cortex of primates. Further, we found that the coding region of female-biased genes is more evolutionarily constrained compared to the coding region of both male-biased and non sex-biased brain expressed genes. We found genes with conserved sexual gene expression dimorphism in the occipital cortex of humans, cynomolgus macaques, and common marmosets. Genes within sexual expression profiles may underlie important functional differences between the sexes, with possible importance during primate evolution.

## Author Summary

The contribution of genetics versus environment to behavioral differences between the sexes is a fundamental question in neuroscience. We hypothesized that some differences between the sexes might be partially explained by sexually dependent gene expression differences in the brain. We further speculated that if differences in gene expression between males and females are functionally important, they may be conserved in the evolution of primates. To test these hypotheses, we measured gene expression in the brains of male and female primates from three species: humans (*Homo sapiens*), macaques (*Macaca fascicularis*), and marmosets (*Callithrix jacchus*). Our results point to a conserved signature of sexual gene expression dimorphism in the brains of primates. Interestingly, we found that genes with conserved sexual gene expression dimorphism in the brain also evolve under more evolutionary constraint, compared with other genes, suggesting that they may have important roles during evolution of sex in primates. Moreover, we found higher evolutionary constrains in the coding regions of female-biased genes as compared to both male-biased and non sex-biased brain expressed genes. The study of sex dimorphic genes may in the future shed light on the basis of psychiatric diseases with differences in prevalence between the sexes.

## Introduction

Many primates are sexually dimorphic in a variety of characteristics including overall body size, tooth dimensions, color and pattern of fur and skeletal features [1]. Additionally, there are behavioral differences between the sexes including reproductive behavior, performance of spatial tasks, domination behavior and aggression [1]–[5]. In apes and old world monkeys such morphological and behavioral differences are often extensive. In contrast, most new world monkeys are more sexually monomorphic [6].

Much less is known about sexual dimorphism in the primate brain. Physical and hormonal dimorphism have been described, including brain size and weight, size of specific anatomical regions, grey and white matter content, and hormonal profiles [7]. However, little information is available about sex differences in gene expression patterns in the brain and their possible functional consequences. Two earlier studies have investigated sex differences in the brains of adult humans and they focused on genes encoded in the sex chromosomes [8] [9]. Their results led to the suggestion that there are only limited sex-biased gene expression in the adult brain [10]. More recently, a genome wide survey was performed in many somatic tissues in mice, and hundreds of genes were found to be sexually dimorphic in whole brain [11].

Since striking physiological differences occur between sexes in specific brain regions [12], and since the cortex is responsible for higher behavioral functions, we decided to investigate specifically this tissue in our studies. Here we present the first genome-wide comparison of sex differences in gene expression in a specific brain region in primates.

We hypothesized that molecular variation in sexual dimorphism may exist in the primate cortex, and that the number of gene expression differences between males and females may reflect this molecular dimorphism. We also speculated that if gene expression differences between the sexes are essential for key male and female characteristics, then these regulatory differences between the sexes may be evolutionary conserved.

In order to determine whether genes are differently expressed in the brains of males and females within primate species, and whether there are any fundamental primate-wide differences in cortex gene expression between the sexes, we compared gene expression levels in the occipital cortex of four male and four female individuals in each of three primate species: humans (*Homo sapiens;* a great ape), macaques (*Macaca fascicularis;* an old world monkey, mainly polygamous [13]), and marmosets (*Callithrix jacchus;* a new world monkey, mainly monogamous [14]). The evolutionary relationships among the primates are illustrated in Figure S1.

## Results

### Microarray Analysis of Sex Differences in Primate Occipital Cortex

**Figure 1:**
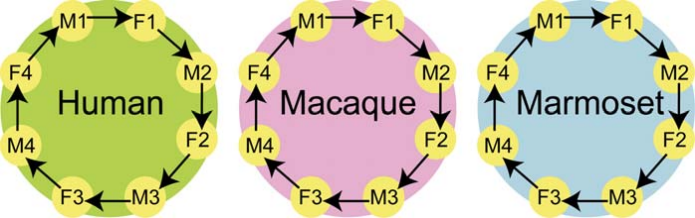
Experimental design. Total RNA was extracted from occipital cortex from four males and four females for each primate species; human (*Homo sapiens*), macaque (*Macaca fascicularis*) and marmoset (*Callithrix jacchus*). Transcribed cDNA (n = 24) was hybridized to human cDNA microarrays (n = 24) (Human 46k cDNA, KTH Microarray Center, Stockholm) in male-female pairs within each species. Yellow circles represent RNA samples from females (F) and males (M). Arrows symbolize microarray hybridizations, where the tip of the arrow indicates that the sample was labeled with Cy5 dye and the base of the arrow that the sample was labeled with Cy3 dye.

To identify genes in occipital cortex that are differentially expressed between the sexes, we hybridized cDNA from each sample (n = 24) of the three primate species to human cDNA microarrays containing probes for 14,621 HUGO annotated genes (KTH Human 46k cDNA, http://www.biotech.kth.se/molbio/microarray/). We used a loop hybridization study design restricted to within-species comparisons, in which we co-hybridized samples from the opposite sex on each array (Figure 1 and Materials and Methods). As expected [15], samples from all species hybridized well to the human cDNA array. This is illustrated by a comparison of overall absolute intensity levels (raw data is available at Array Express database under accession E-MEXP-1182). Since we used cDNA microarrays and performed all competitive hybridizations between females and males from the same species, our results are not expected to be biased by the effect of sequence mismatches on hybridization intensity [15].

### Extent of Sexual Gene Expression Dimorphism in Occipital Cortex in Human, Macaque, and Marmoset

To determine the degree of sexual dimorphism within each of the three primates, the data from each species was analyzed independently and genes expressed differently between the sexes were identified. We applied single channel normalization [16] to acquire absolute intensities for each clone and individual sample. To identify genes that are differentially expressed between the sexes, we used a linear model to analyze gene specific expression levels from each species, with the penalized F-ratio (PenF) for a sex difference as our ranking statistic (see Materials and Methods for details). By this approach, the analyzed genes in each species were ranked according to the size and reproducibility of the expression difference (Table S1).

**Figure 2:**
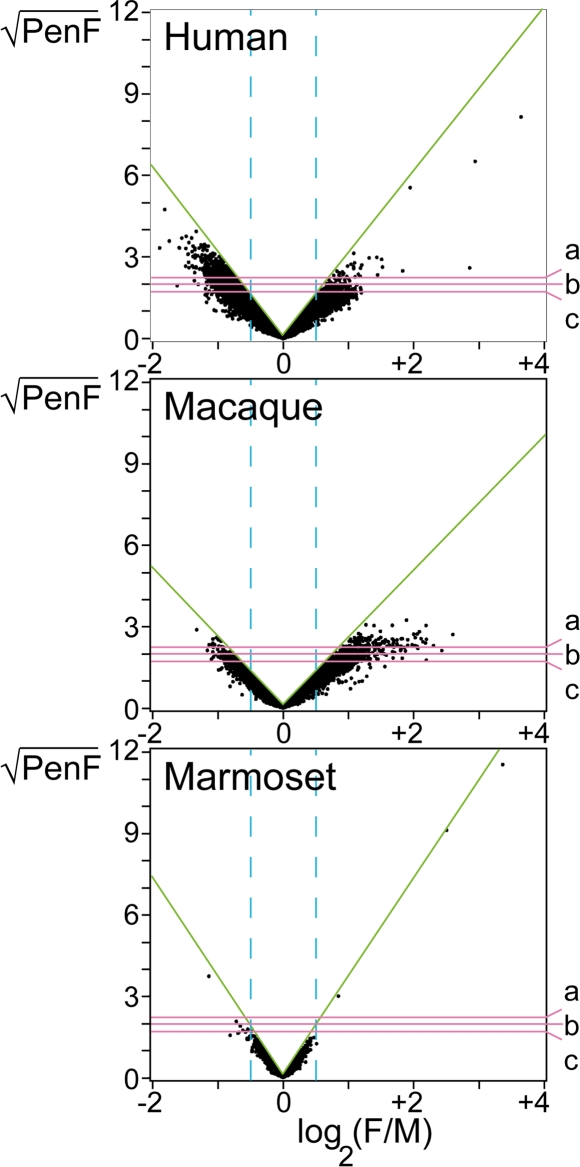
Volcano-plots of cDNA microarray data in three primate species. The figure shows an overview of the microarray results in each of the three primate species; human (*Homo sapiens*), macaque (*Macaca fascicularis*), marmoset (*Callithrix jacchus*). The y-axis denotes √(PenF) i.e. significance level, and the x-axis denotes log_2_(F/M) i.e. fold change expression difference between females and males. The horizontal lines; a, b, c, illustrate the PenF thresholds corresponding to Table 1. The vertical lines represent log_2_ fold changes of 0.5 (i.e. 1.4 on decimal base), marked to facilitate comparisons between the three graphs. The green lines tangent the maximum √(PenF) for any given fold change. The slope of this line is inversely proportional to √a0 (the penalty constant), and a gentle slope is reflecting a relatively high technical and biological variation.

We found several hundreds of genes (Table 1) to be differentially expressed in the occipital cortex of males and females in human and macaque, while fewer than ten were sexually dimorphic in marmoset. Table 1 lists the number of clones above three threshold values of the ranking statistic (PenF: 3.0, 4.0 and 5.0) in each species, along with an estimated false discovery rate (FDR) associated with the threshold. The volcano-plots in Figure 2 show an overview of the data for each species with the thresholds corresponding to Table 1 represented by three horizontal lines. The differences in population of sex-biased genes between marmoset and the catarrhine species is striking.

**Table 1:** Number of sexually dimorphic genes in occipital cortex of human, macaque and marmoset.

	PenF		
	3.0^a^	4.0^b^	5.0^c^
**Human**	1349	705	407
FDR	0.08	0.05	0.04
**Macaque**	486	224	116
FDR	0.25	0.17	0.15
**Marmoset**	7	5	4
FDR	1.00	1.00	0.00

The table shows the number of clones with PenF thresholds of 3.0, 4.0 and 5.0 in each primate species; human (*Homo sapiens*), macaque (*Macaca fascicularis*) and marmoset (*Callithrix jacchus*), along with an estimated false discovery rate (FDR) associated to each PenF value. The letters a, b, c, above the PenF values correspond to the illustrated thresholds in Figure 2.

**Figure 3:**
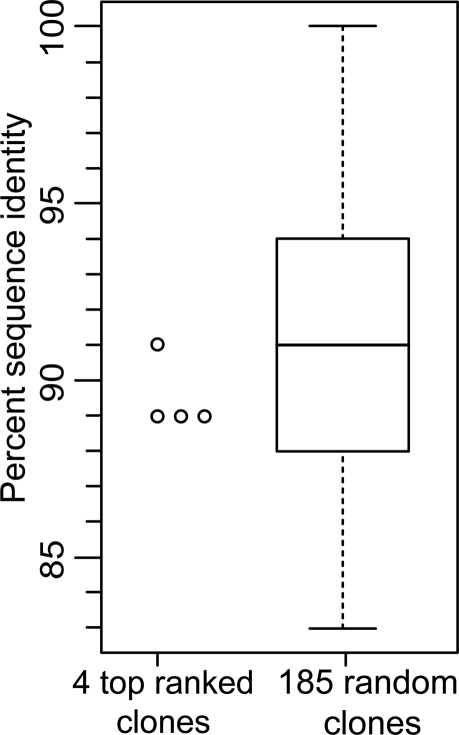
Sequence identity between selected marmoset transcripts and their orthologous human cDNA clones present on the microarrays. The figure shows that the 4 highest ranked clones with respect to sexual dimorphism in marmoset (*Callithrix jacchus*, PenF≥5.0, Table 1 and Figure 2) do not have a higher sequence identity distribution than a random sample of low ranked genes (n = 185). This demonstrates that the low number of sexually dimorphic genes in marmoset is not mainly due to an effect of sequence identity difference between the species.

The observation of a few prominent genes in marmoset, having both high PenF and fold change values, also indicates that reaching a detectable signal level in this species was not a concern. However, a caveat to our analysis is that sequence mismatches could result in reduced power to detect differentially expressed genes using cross-species hybridizations [17]. To investigate this issue, we studied sequence identity between marmoset genes and human cDNA clones present on the microarrays. Sequence identities between human cDNAs on the microarray and marmoset genes were calculated (see Materials and Methods). As shown in Figure 3, the 4 highest ranked clones in marmoset (Table 1 and Figure 2) do not have higher sequence identity than a random sample (n = 185) of low ranked genes. We conclude that sequence divergence did not seriously contribute to the exceptional differences in numbers of sexually dimorphic genes between marmoset and the catarrhines. However, an effect of sequence divergence on some sexually dimorphic genes in each species cannot be ruled out.

### An Evolutionary Conserved Sexual Signature in Primate Occipital Cortex

In order to identify possibly conserved sexual expression differences in the primate occipital cortex, the differences between the sexes in samples from all combinations of species was determined. To do this, the gene specific expression levels of 16 individuals at a time (for two species comparisons), or all 24 individuals at a time (for three species comparisons), were analyzed simultaneously with the linear model used to analyze single species, adding the effect for species and the interaction effect between species and sex. Conserved sexually differentiated genes were defined as genes with a large (and reproducible) average difference over the analyzed species, and a relatively small difference in sexual dimorphism between species (see Materials and Methods for details).

**Figure 4:**
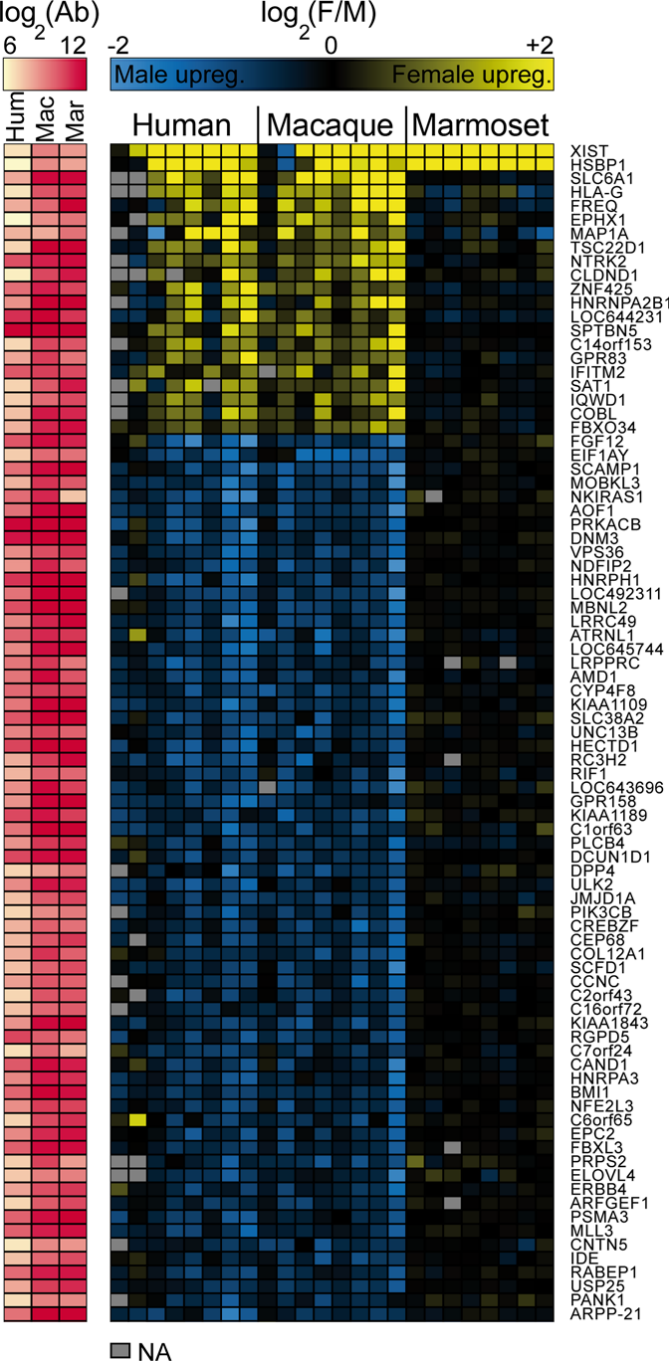
A conserved sex signature in occipital cortex gene expression among human, macaque and marmoset. The presence of a conserved sex signature of occipital cortex gene expression in human (*Homo sapiens*), macaque (*Macaca fascicularis*) and marmoset (*Callithrix jacchus*) is here shown by a heatmap. Rows represent genes and each column a male Vs. female hybridization. Yellow illustrates female up-regulation, blue illustrates male up-regulation, and black illustrates no difference in gene expression between the sexes. The red map on the left shows background subtracted mean intensity values (Ab) for each gene and species. The intensities in marmoset are not lower than in the other species, demonstrating that the lack of sex differences in marmoset was not due to weak binding to the human cDNA microarrays. The two top genes (*XIST*, *HSBP1*) display conserved sexually dimorphic expression patterns among all three species while the following 83 genes display a conserved sex-specific expression pattern in humans and macaques. Selection criteria: FDR≤0.05.

Using this approach, we identified 85 genes with sexually dimorphic expression profiles in the same direction in both humans and macaques (FDR≤0.05, Figure 4). This provides the first observation of conserved sexually dimorphic gene expression signature in primate brains. Further, 2 genes, X inactivation-specific transcript (XIST) and Heat shock factor binding protein 1 (HSBP1), were consistently sex-biased in all three species (FDR ≤0.05, Figure 4), both of these genes were upregulated in females. These two genes were also the only genes identified in the combinations human-marmoset and macaque-marmoset. Figure 4 also shows that absolute intensities for marmoset samples were not lower than intensities for the other species (left panel red color), indicating that hybridization intensities were not biased due to an effect of sequence mismatches between primate RNA samples and human cDNA probes on the microarrays.

### Expression Patterns of Genes in the Conserved Sexual Signature in Human Tissues

**Figure 5:**
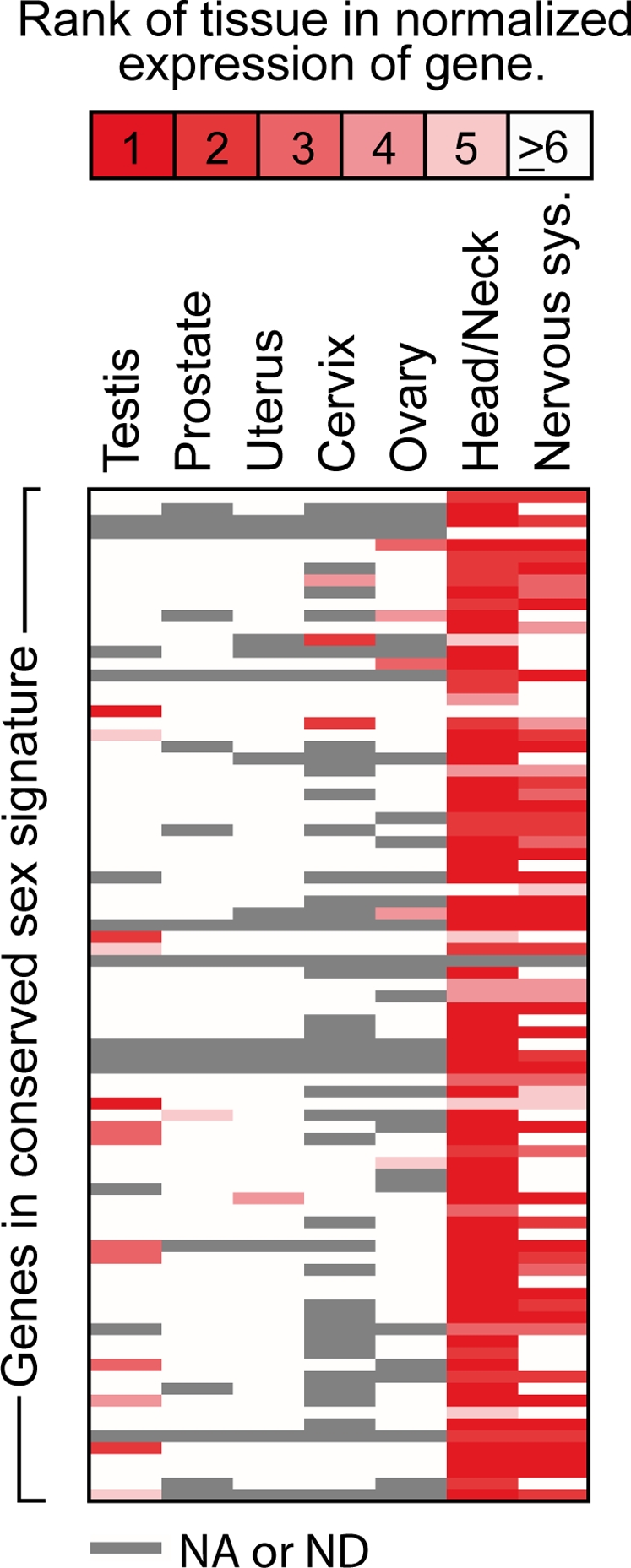
Expression of the occipital cortex conserved sexually dimorphic genes in other human tissues. Many of the genes in the identified conserved sex signature in primate occipital cortex are highly expressed in the nervous system, and expressed less in sexual tissues. To generate the figure, we used normalized expression data for human tissues from SOURCE [19]. Normalized gene expression presents the relative expression level of a gene (defined as a UniGene Cluster) in different tissues and is normalized for the number of clones from each tissue that are included in UniGene. Rows denote genes and columns denote tissues. Rank 1 means that expression of a gene is highest in the indicated tissue, symbolized by deep red color, and lower ranks are represented by steps of less intense red, as indicated by the color code bar at the top of the figure. Gray color denotes that expression level of the gene in tissue was not available or not detected (NA or ND). 55 out of the 85 genes in the conserved sex signature have a rank equal to or higher than 5 in nervous tissues (65%). 81 of the occipital cortex sex signature genes are highly expressed in tissues present in the head or neck (95%, rank ≥5), while 23 of the genes are highly expressed in any of the sexual tissues (27%, rank ≥5). Genes appear in the same order as in Figure 4.

It is possible that the genes that were identified as conserved sex-biased in cortex of human and the other primates are sex-biased not only specifically in brain, but also in other tissues. It is also possible that the sex-bias observed is more the result of selective constraints that operate on other tissues as opposed to brain. Many genes that are expressed in gonad tissues are sex-biased [18], and we therefore investigated if the genes in the conserved sex signature in primate occipital are highly expressed in sexual tissues and/or in nervous tissue. We used publicly available expression data for human tissues from SOURCE [19], where tissues are ranked according to their normalized expression of each gene (see Materials and Methods for details). We found that a majority of the 85 genes in the conserved sex signature, 55 (65%), had nervous tissues ranked equal to or higher than 5 (Figure 5), and that only 23 genes, (27%) were highly expressed in sexual tissues.

These results show that many genes in the conserved signature of sex-biased expression are highly expressed and are therefore likely to be functional in the nervous system. Since a smaller fraction of the genes are expressed highly in sexual tissues it is possible that selection of sex-biased expression of many of the genes has been on the nervous system (or other tissues) rather than on sexual tissues.

### Analysis of Non-Synonymous versus Synonymous Substitution Rates in Sexually Dimorphic Genes in the Primate Occipital Cortex

**Figure 6:**
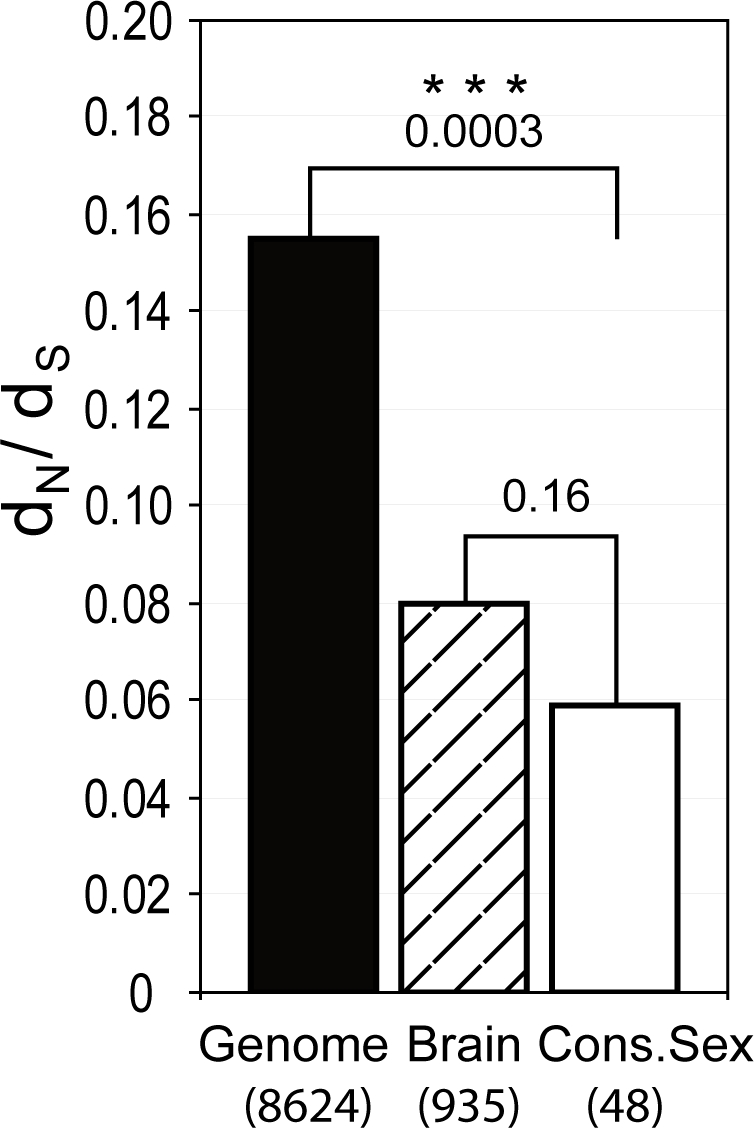
Non-synonymous versus synonymous substitutions in conserved sexually dimorphic genes. We estimated non-synonymous and synonymous ratios (d_N_/d_S_ ratios) in a large set of human-chimp-rhesus three-way alignments. The d_N_/d_S_ ratios of genes in the conserved sexual signature in humans and macaques (genes in Figure 4, Cons.Sex, n = 48 found in the three-way alignment) were compared to genes specifically expressed in brain [20] (Brain, n = 935), and to all other genes found in the three-way alignment (Genome, n = 8624). The genes in the conserved sex signature had d_N_/d_S_ ratios significantly lower than the genome reference (median of Cons.Sex = 0.06, median of Genome = 0.16, permutation test of the medians; p = 0.0003), and d_N_/d_S_ ratios in the same range as other brain expressed genes (median = 0.08). The heights of the bars represent median d_N_/d_S_ ratios. p-values; *** p≤0.001.

We hypothesized that if the conserved signature of sexually dimorphic gene regulation in the brains of human and macaque is functionally important, the genes included in this signature may also be more conserved at the protein level. To test this, we studied the rate of protein evolution in the human lineage by estimating the ratio of the rates of amino acid changing (non-synonymous, d_N_) to silent (synonymous, d_S_) substitutions (d_N_/d_S_) (see Materials and Methods for details). As can be seen in Figure 6, we found that d_N_/d_S_ ratios for genes that show conserved sexually dimorphic expression profiles are significantly lower (median = 0.06) compared to d_N_/d_S_ ratios of other human genes (median = 0.16) (permutation test for the difference between medians; p = 0.0003). However, genes expressed in brain are known to be under strong evolutionary constraint [20] and we therefore also compared the conserved sex signature with human brain-expressed genes only. We find a tendency, although not statistically significant, of higher conservation of sexually dimorphic genes when comparing them with the brain-expressed genes in general (median = 0.08).

### Analysis of Non-Synonymous versus Synonymous Substitution Rates in Male-Biased and Female-Biased Genes

**Figure 7:**
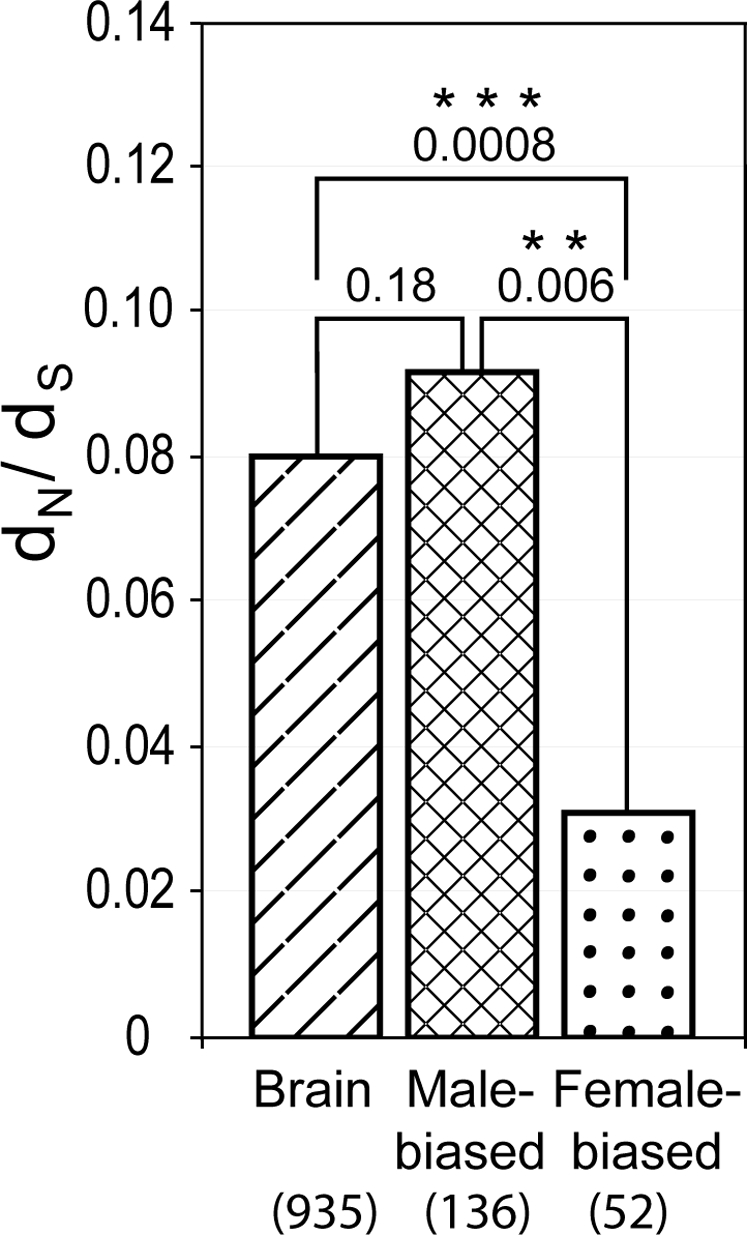
Non-synonymous versus synonymous substitutions in male-biased and female-biased genes. Using a large set of human-chimp-rhesus three-way alignments, we compared ratios of non-synonymous versus synonymous substitutions (d_N_/d_S_) between genes that were identified as male up-regulated (Male-biased) and female up-regulated (Female-biased) in human and/or macaque in the single species analysis (genes with PenF≥5, Table 1 and Figure 2). 136 of the male-biased genes and 52 of the female-biased genes were found in the three-way alignments. Male-biased genes had d_N_/d_S_ ratios three times higher than female-biased genes (median of male-biased = 0.09, median of female biased = 0.03, permutation test of the medians; p = 0.006). Non-biased brain-expressed genes had intermediate d_N_/d_S_ ratios (median = 0.08). The heights of the bars represent median d_N_/d_S_ ratios. p-values; ** p≤0.01, *** p≤0.001.

Genes with male-biased expression are expected to have higher d_N_/d_S_ ratios than female-biased genes [21]. We investigated whether this holds true for genes recognized as sex-biased in our primate dataset. We compared d_N_/d_S_ ratios between genes that were identified as male-biased and female-biased in human and/or macaque in the single species analysis (genes with PenF≥5.0, Table 1, Figure 2, Table S1). We found d_N_/d_S_ ratios of male-biased genes to be three times higher than d_N_/d_S_ ratios of female-biased genes (Figure 7, median of male-biased = 0.09, median of female-biased = 0.03, permutation test of medians; p = 0.006). When comparing this data with a set of genes that are non-sexually-biased (the brain-expressed genes used in Figure 6), we found that non-biased brain expressed genes have intermediate d_N_/d_S_ values (median = 0.08). This is interesting, since the pattern seen here in primates has earlier been observed in *Drosophila* [22].

### Evaluation of Genes in the Conserved Sex Signature Using RT q-PCR

**Figure 8:**
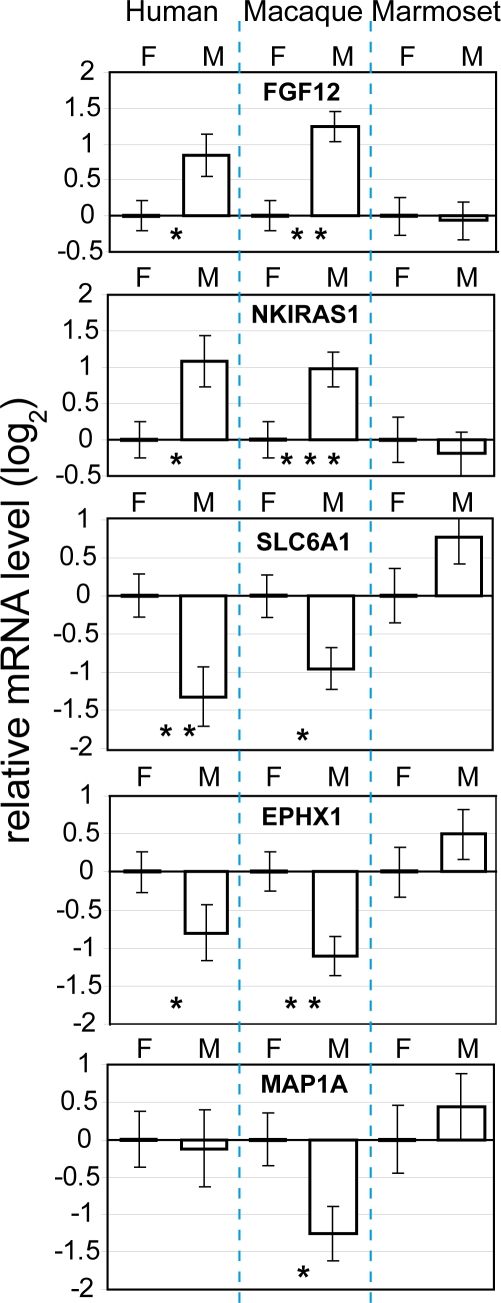
Confirmation of conserved sex expression signature with RT q-PCR. mRNA levels of two genes with male-biased expression and three genes with female-biased expression in human (*Homo sapiens*) and macaque (*Macaca fascicularis*), but not in marmoset (*Callithrix jacchus*), as quantified with RT q-PCR. The expression patterns observed in the array experiment (Figure 4) were confirmed for each gene, with the exception of MAP1A, which was not differentially expressed between males and females in human. Least square means and standard errors of the mean are given. Female expression levels were used as point of reference. p-values; * p≤0.05, ** p≤0.01, *** p≤0.001.

We evaluated three male-biased and three female-biased genes in the conserved sex signature using RT q-PCR in order to confirm the microarray results. Primer pairs were designed on conserved regions, forward primer and reverse primer located on different exons, for the following genes: FGF12, NKIRAS1, AOF1, SLC6A1, EPHX1, MAP1A (Table S2). We measured transcript levels in four males and four females in each of the three primate species. We employed non-template controls (NTCs) for each individual sample and gene to control for DNA contaminations. Actin-β (ACTB) was used as reference transcript (see Materials and Methods for details). Transcript levels in five of the six transcripts could be quantified; FGF12, NKIRAS1, SLC6A1, EPHX1, MAP1A (Figure 8). Contributions from DNA contaminations were negligible. Typical cycle threshold values (CT) of the NTCs were at least 15–20 PCR cycles higher than those of cDNA samples. AOF1 could not be quantified (NTCs in the same ranges as cDNA samples). The expression patterns in species and sex could be statistically confirmed and were consistent with the microarray results for each of the five quantified genes except for MAP1A, in which we did not find a difference between male and female expression in human.

### Analysis of Biological Categories in the Conserved Sexually Dimorphic Genes

To evaluate whether certain biological categories are overrepresented among the conserved sexually dimorphic genes shown in Figure 4, we categorized the genes according to their functional annotations (*DAVID* [23]). We then assessed overrepresented ontology classes in the conserved sex-biased genes compared to the genes on the microarray overall. The choice of reference set in analysis of overrepresented gene ontology classes can affect the results. We therefore also compared the conserved sex-biased genes with the 50% of genes on the microarray with highest expression (i.e. highest intensity values). In Table 2, we report overrepresented ontology classes with p-values consistently lower or equal to 0.05, using both reference sets. Notably genes involved in metabolism, polyamine biosynthesis and ubiquitin cycle, were overrepresented.

**Table 2:** Overrepresented ontology classes in the conserved sex signature in primate occipital cortex.

				Cons.Sex Vs. All genes on Array		Cons.Sex Vs. 50 % highest expressed	
Category	Term	Gene Count	%	p-value	Fold Change	p-value	Fold Change
GENETIC_ASSOCIATION_DB	METABOLIC	4	4.7%	0.01	4	0.05	5
SP_PIR_KEYWORDS	polyamine biosynthesis	2	2.4%	0.03	76	0.04	52
COG_KOG_ONTOLOGY	General function prediction only	7	8.2%	0.03	2	0.02	2
GOTERM_BP_ALL	ubiquitin cycle	7	8.2%	0.04	3	0.04	3

The table shows overrepresented ontology classes in the conserved sex signature (85 genes in Figure 4) obtained after GO analysis [23]. Only terms with p-values consistently lower than or equal to 0.05, using two different reference sets (all genes on the microarray and the 50% of genes on the microarray with highest intensities) are shown. % indicates fraction of genes in the conserved sex signature belonging to each ontology class.

### Prediction of Conserved Estrogen and Androgen Cis-Response Elements in the Conserved Sexually Dimorphic Genes

Sex hormones, such as estrogens and androgens, have been considered to have great influence on the differences between the male- and the female brain [24]. As a first step, to identify potential conservation of sex hormonal regulation, we decided to investigate the presence of estrogen- and androgen response elements in genes with conserved sexual dimorphic expression in the two catarrhine species. A search for estrogen alpha and androgen cis-response elements (ESR1 and AR) was performed in genes within the conserved sexual expression signature in human and macaque (genes in Figure 4). Of these 85 genes, 61 orthologs genes pairs could be identified in the two species. Sequences from the closely related species *Macaca mulatta* available in Ensembl (http://ensembl.org) were used instead of *Macaca fascicularis*. Prometheus system [25] was used to analyze the presence of ESR1 and AR in the regions; 10000 bases upstream the transcription initiation site, introns and 5000 bases downstream the 3′ end of the genes. The transcription factor binding sites were identified using weight matrices from JASPAR Database [25]. Only regions with 95% of sequences similarity between human and macaque were considered in the analysis. The threshold for positive identification of binding sites based on the matrix model similarity was 80%. We find that 34 (56%) of the 61 genes contained at least one ESR1, and 40 (66%) contained at least one AR. 16 (26%) of the 61 genes contained neither ESR1 nor AR in the investigated regions. Table S3 contains the annotations of the 61 genes investigated here and the locations of ESR1 and AR elements in these genes.

## Discussion

We identified genes with conserved sex differences in mRNA expression in occipital cortex among three primates: human (*Homo sapiens*), macaque (*Macaca fascicularis*) and marmoset (*Callithrix jacchus*). This finding establishes the existence of biological sex differences in gene expression the human cortex, and further, it unveils the existence of conserved sexual signatures in the primate cortex with possible importance during primate evolution. The obvious question to follow is whether or not these signatures of sex in the brain have physiological significance for brain physiology and/or behavior. In *Drosophila*, sex-dependent selection may drive changes in expression of many of the most rapidly evolving genes and sex specific transcriptome differences may be the driving force behind speciation events [26] [27]. This example shows that molecular sex differences may be important during evolution because sex-specific genes may be subjected to different selective pressures on each sex. Not only the fruit fly, but also many other species including many primates show great morphological sexual dimorphism. Physical dimorphism goes along with social and behavioral dimorphism, which may be reflected in gene expression in the brains of these species. Variation in brain gene expression has been observed even between closely related species [28]. Our results suggest that variation in expression of genes in the brain may be an important component of behavioral variation within as well as between species. Conservation of gene expression patterns alone may be inadequate to suggest that genes included in the conserved signature are important for physiological differences between the sexes in the brain. However, when estimating non-synonymous and synonymous substitution rates (d_N_/d_S_ ratios) in the coding region of the genes in the conserved sexual expression signature, we find that these genes evolve under more selective constraint in the human lineage compared with a genome-wide control-set of genes (p = 0.0003). Brain expressed genes are known to be under strong evolutionary constraint [20]. We find a tendency of lower d_N_/d_S_ ratios even when comparing to other brain genes, although not statistically significant (p = 0.16). This observation is consistent with the hypothesis that these genes have important function(s) in the brain [29], possibly of fundamental importance to sex differentiation.

An observation that reinforces this hypothesis is that most of the genes included in the conserved signature are highly expressed in nervous tissues (65%), while fewer are expressed highly in sexual tissues (27%), as shown in Figure 5. It is therefore probable that the sex differences in expression in the brain are not just a reflection of their functional significance in gonads, but may suggest that they are physiologically relevant for sex differences in the brain itself. It is consequently possible that selection of sex-biased expression of many of the genes that were identified has been on the nervous system (or other tissues) rather than on sexual tissues.

Also important for evolutionary discussions, we observed among the sex-biased genes that are not conserved during evolution a significantly higher constraint in evolutionary rates of coding sequences in female-biased genes as compared to male-biased and non-biased genes (Figure 7). Interestingly, the same pattern has recently been observed in a large study of sexually dimorphic gene expression in *Drosophila* [22]. This could suggest that the female-biased genes are subject to stronger purifying selection than the male-biased genes, which corresponds well with the idea that natural selection is more important than sexual selection in female mammals. This pattern could also or alternatively be explained by positive selection in the male biased genes [26], which may be more exposed to labile and species specific sexual selection.

We do not know what regulatory mechanisms are controling the sexually dimorphic expression of the genes that were identified in our study. It is possible that the expression of some of these genes is under sex hormonal regulation, but this is yet to be decided. As a first step to identify potential conservation of sex hormonal regulation, we investigated the presence of estrogen alpha- and androgen response elements in conserved regions in human and macaque in the genes identified in out study. The results are presented in Table S3. It is too early to make any general statements based on this data and further studies in these issues should be done.

To avoid the effect of sequence divergence on the evaluation of sex differences in gene expression [17], we restricted the microarray analysis to intra-species comparisons between the sexes. This approach is valid in confirming the conservation of expression differences during evolution, since the effect of sequence deviation between probes on the microarray and the primate RNA should be identical in males and females of the same species. On the other hand, the strategy has limitations. First, because of sequence divergence it is possible that some differently expressed genes were not identified in marmoset and/or macaque. For this reason, it is possible that the conserved cortex sex dimorphism across primates may actually be more extensive than here described. The method may also be imperfect in its ability to investigate divergence of sex differences between humans and the other primates because of the effect of sequence divergence. Nevertheless, the comprehensive dissimilarity in the total number of genes with sexually dimorphic expression in the marmoset occipital cortex (7 clones) compared to that of human (1349 clones) and macaque (486 clones) (PenF≥3.0, Table 1, Figure 2) is substantial enough to suggest that there may be essential biological differences in the prevalence of sex differences in the brain of marmosets compared to that observed in the two strongly sexually dimorphic species. Moreover, the sequence comparisons and the strength with which all species hybridized to the microarrays indicate that sequence divergence does not explain the extreme differences in the number of dimorphic genes between these species. However, sequence divergence cannot be totally ruled out as an underlying factor. The observation of minute sex differences in the marmoset brain is interesting since it correlates with the overall trend in the marmosets, which are relatively sexually monomorphic in their physical constitution as compared to the more strongly sexually dimorphic humans and macaques [6]. In primates, a general rule is that many of the sexual physical and behavioral dimorphisms that are so pronounced in the polygamous species, such as macaques, are typically less distinct or lacking in monogamous species, such as the marmosets [1],[14]. Our observation suggests that this correlation may also be reflected in the degree of sex-biased gene expression in the brain.

Two sexually dimorphic genes were conserved across all three of the primates, suggesting that these genes may have essential functions related to key male and female characteristics. One of these genes is on a sex chromosome (*XIST* on the X chromosome) and is key in the inactivation of one of the two X chromosomes in mammalian females. The second is an autosomal heat shock binding protein (*HSBP1* on chromosome 16*)* that has been shown to bind and negatively regulate heat shock factor 1 (*HSF1*) [30], which in turn is known to be regulated by estrogen [31]. The *HSBP1* gene is important for regulating the physiological reactions to stress [30], raising the intriguing possibility of a conserved sexual difference in stress response in primates [32]–[34].

In conclusion, our observations suggest that some sexual differences in the occipital cortex at the gene expression level may be conserved during the evolution of primates. Multiple lines of research have observed sex differences in behavioral and cognitive abilities in humans [35]–[37] and other primates [4],[5],[38]. However, whether these differences are caused by biological changes present in the brain is not yet known. The study of sexual differences in gene expression in the primate brain is important not only to increase our understanding of sex differences in normal behavior, but also to explain differences between the sexes in prevalence of psychiatric diseases and response to drug treatments [39]. Our findings should thus fuel future investigations on the precise role of sexually distinct expression profiles and their possible involvement in physiology, behavior and cognition.

## Materials and Methods

### Tissue Samples

Tissues were obtained from the occipital cortex from macaques, marmosets and humans. Four males and four females from each species were included in the experiments. The brain samples from macaques (*Macaca fascicularis*) were controls from a previous research program conducted by Dr. Diana Radu at the Karolinska Institute, Stockholm. The animals had been housed at The Swedish Institute for Infectious Disease Control, in Stockholm. The marmoset (*Callithrix jacchus*) samples were a donation from IMANET, Uppsala, where the animals had been used in the development of PetScan analyses and they were housed at the facility for laboratory animals, Uppsala University. The human samples were previously described occipital cortex control samples [40]. The brains were stored at −70°C prior to analysis. Occipital cortex was dissected by applying a vertical cut at the back of the frozen brains and splinters of cerebellum were separated from the cortex slices with a scalpel, keeping the samples at all times on dry ice and inspecting the brain structures with a magnifying glass. Weights of samples were in the range of 0.28–0.42 g.

### Isolation of Nucleic Acids

Nucleic acids were extracted from frozen tissues which were homogenized in TRIzol and RNA was extracted according to the manufacturers' instructions (Invitrogen™, Life Technologies) as in [40],[41]. The concentration of extracted RNA was measured using a NanoDrop ND-1000 instrument (NanoDrop Technologies, USA) and ranged between 2860–5870 ng/µl for macaques, 3420–4330 ng/µl for marmoset and 1900–3240 ng/µl for humans. Electrophoresis (1% agarose, 0.5 x Tris-Acetate-EDTA buffer, 90 V, 30 min, 6 µl sample, 3 µl 3 x loading buffer) was run for RNA quality inspection. The gel was stained in an ethidium bromide bath.

### Labeling and Microarray Hybridizations

30 µg of total RNA from each male and female sample were reversely transcribed to cDNA with Cy3/Cy5-3DNA catch sequences incorporated in the primers, using Genisphere 3DNA Array 900 labeling kit (Genisphere Inc., USA) and Superscript II RT enzyme (Invitrogen, Life Technologies) with supplied buffers and following the manufacturers' instructions. cDNA was concentrated in Microcon YM-30 columns (Micron Bioseparations Millipore Corporation). The microarray experiments were set up in a loop design (Figure 1). Each individual sample was hybridized twice, each time labeled with a different fluorescent dye (Cy3 or Cy5) and coupled with a sample of the opposite sex. This design resulted in a total of 24 co-hybridizations (eight hybridizations for each of three species).

Hybridizations were performed on preheated microarray slides printed with 46,128 cDNA clones (KTH Human 46k Batch11, Microarray Resource Centre, Royal Institute of Technology, Sweden), using the buffers and procedures given in the Genisphere 3DNA Array 900 labeling kit. Hybridizations were conducted in humidity chambers (Corning Inc.) at 42°C for 18 h. The microarray slides were subsequently washed in glass slide holders shaking at 320 rpm: 2 min in 2 x sodium chloride-sodium citrate buffer (SSC), 0.2% sodium dodecyl sulfate (SDS) at 65°C; 10 min in fresh 2× SSC, 0.2% SDS at 65°C; 10 min in 2× SSC at room temperature; 10 min in 0.2× SSC at room temperature. Arrays were dried by centrifugation at 1000× g. Arrays, cover slips and humidity chambers were preheated and 3DNA Cy3/Cy5 hybridization mix added onto the arrays in accordance with the Genisphere protocol. 3DNA-hybridization was conducted in humidity chambers at 42°C for 5 h. The slides were washed again as described above, but with an additional final wash for 5 min in 0.1× SSC at room temperature prior to centrifugation and scanning.

### Microarray Analysis

The 24 microarrays were scanned at 10 µm resolution using a GenePix 4100A scanner (Axon Instruments Inc.). Spots on the resulting images were quantified with the software package GenePix Pro 5.0 (Axon Instruments Inc.). No background correction was applied to the data. However intensity values after mean background subtraction was calculated to evaluate the quality of each array (Figure S3). The resulting files are publicly available on the EMBL-EBI ArrayExpress database (ArrayExpress accession E-MEXP-1182, conforming to the MIAME guidelines).

Analysis was done with SAS software (SAS/STAT, version 9.1.3, SAS institute Inc., Cary, NC). The log2 transformed mean intensities of Cy5 (R) and Cy3 (G) for each spot were used to calculate the log-transformed ratio M = R-G. A robust scatter plot smoother (Proc Loess, SAS version 8.02) was used to remove systematic intensity dependent dye-bias from M. This within slide normalization was done for 12 sub-array blocks separetly giving Msa, with the smoothing parameter set to 40% (Figure S2). Next, quantile normalization [42] was used to adjust the signal intensities between slides (within species). We applied quantile normalization to the intensity A = ½(R+G), giving Aq. The fully normalized R and G values were then defined as: RMsaAq = ½(Msa+2Aq), GMsaAq = ½(Msa-2Aq) [16]. All spots with a mean spot intensity below the local median background were excluded from subsequent analysis.

To further evaluate the quality of the data from all arrays, we plotted the average of the background subtracted intensity values for each array versus the number of missing spots for that array. We noted that the arrays that included a human female called “F4” were of low quality compared to all other arrays, yielding 50.2% and 35.6% of missing probes while the average for all the other 22 arrays was 6.4%, and having lower mean spot intensity values (Figure S3). Therefore, this individual was removed from all further analysis. However, the data from this individual is still shown in Figure 4, for completeness.

### Statistical Analysis of Microarray Data

In order to identify genes that are differentially expressed between the sexes we determined whether the average expression difference over the four males and females was significant compared to inter-individual variation for each gene. This was done by analyzing the microarray data with the following mixed linear model (single channel analysis) with Procedure Mixed (n_ind_ = 8, n_y_ = 16),
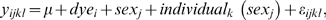
in which the gene labels have been suppressed. Here y_ijkl_ is the log transformed and normalized intensity (R_MsaAq_ or G_MsaAq_) of the *l:*th replicate of the *k:*th individual of sex *j*, incorporated with dye *i*. Dye and sex were considered fixed factors, where as individual was considered a random factor.

Differentially expressed genes were ranked by the F-ratio for the factor sex, which was penalized by adding a constant (a_0_) to the denominator. We chose a_0_ to be the 90^th^ percentile of the mean square for individual of all analyzed genes. A false discovery rate (FDR) for each species list was determined empirically for a range of penalized F-ratios (PenF), by permuting sex within species. P-values were defined as the fraction of simulations that yielded at least the number of observed genes. For each species there were 35 unique permutations, (_7_C_4_ for human, and _8_C_4_/2 for macaque and marmoset), yielding a minimum p-value of 0.029.

In order to identify conserved sex expression differences across primate species, we combined samples from two respectively all three species. To do this, the gene specific expression levels of 16 individuals at a time (for two species comparisons), or all 24 individuals at a time (for three species comparisons), were analyzed with the following mixed linear model,
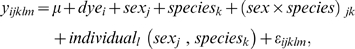
where the annotation is the same as for the single species analysis. Two additional fixed factors, species and sex*species, were included in the model to account for systematic variation between species and to estimate the difference in sexual dimorphism between species respectively. Conserved sexually differentiated genes were defined as genes with a) a large average difference over the analyzed species, and b) a relatively small difference in sexual dimorphism between species. According to this working definition, genes were ranked after their penalized F-ratio for sex (as described above), under the constraint that the mean squares (MS) of sex should be considerably larger than the mean squares for the interaction between sex and species. We chose threshold level of 16 for the ratio between MS_sex_ and MS_sex*species_, which in the two species analysis corresponds to the average sex effect being at least twice as large as the difference in response between the species, (on a logarithmic scale). The FDR for each 2 (or 3) species list was determined empirically for a range of penalized F-ratios for sex, by permuting sex within species. From all possible permutations 1,000 random permutations were drawn. Following the permutations, we selected lists of genes with no more than 5% false discovery rates and these genes were used to construct Figure 4. The figure shows the resulting genes from both the two species and the three species comparisons. The software MeV [43] was used to generate the heatmap.

### Sequence Divergence Analysis

To estimate sequence divergence between marmoset genes and cDNA clones on the microarray, we utilized the nearly complete *Callithrix jacchus* assembly; The Genome Sequencing Center, Washington University Medical School (http://genome.wustl.edu/pub/organism/Primates/Callithrix_jacchus/assembly/Callithrix_jacchus-2.0.2/). Clone EST sequences were used as baits, and Stand-alone BLAST, (NCBI, ftp://ftp.ncbi.nlm.nih.gov/blast/executables/, expectation value (E) set to 10^−6^) was used to obtain marmoset sequences. Identity values in Figure 3 are presented as calculated by the Stand-alone BLAST software.

### Expression of the Conserved Sexually Dimorphic Genes in Other Human Tissues

We investigated if the genes in the conserved sex signature in primate occipital are highly expressed in sexual tissues and/or nervous tissue. We used publicly available expression data for human tissues from SOURCE [19], where tissues are ranked according to their normalized expression of each gene. Normalized gene expression presents the relative expression level of a gene (defined as a UniGene Cluster) in different tissues and is normalized for the number of clones from each tissue that are included in UniGene. Tissues on SOURCE are ranked according to their normalized expression. Tissues with ranks ≥5 were considered in Figure 5. Tissues associated to nervous system were: whole brain, spinal cord, nerve, ganglia, pineal gland and pituitary gland. The software MeV [43] was used to generate the heatmap.

### Analysis of Non-Synonymous versus Synonymous Substitution Rates

In order to test the hypothesis that genes differentially expressed between males and females are under more selective constraint than other genes, we estimated non-synonymous and synonymous substitution rates (d_N_/d_S_ ratios) in a large set of human-chimp-rhesus three-way alignments. These alignments were used in a recent analysis of the Rhesus macaque genome [29] and were kindly provided by Adam Siepel at Cornell University. d_N_/d_S_ estimates were calculated using PAML [44], with the default parameters for nuclear DNA.

The genes were divided into three distinct categories: conserved sexually dimorphic (genes in Figure 4, 48 of the 85 genes were found in the three-way alignment), expressed in the brain (935 genes [20], genes in the brain expressed data set that were also among the conserved sexually dimorphic genes or among the genes identified as sex-biased in the single species analysis at PenF≥5.0 were removed from this group), and all other genes found in the three way alignments (8624 genes). To test whether the medians of d_N_/d_S_ differed between each pair of groups we used a permutation test on the difference between the two medians (*D*). Specifically, we randomly divided all the values into two groups of same sizes as observed, and calculated the medians of the random groups. This permutation was repeated 10,000 times, and each time the difference between the medians of the two randomly selected groups (*D*_i_) was recorded. The test p-value was defined as the number of times where *D*_i_≥*D*, divided by 10,000.

In the analysis of male-biased and female-biased d_N_/d_S_ ratios, we used genes that were identified as sexually dimorphic in human and/or macaque in the single species analysis (genes with PenF≥5.0, Table 1, Figure 2, Table S1). Overlapping probes were removed from the analysis. 136 of the male-biased genes and 52 of the female-biased genes were found in the three-way alignments.

### Evaluation of Genes in the Conserved Sex Signature Using RT q-PCR

Primers were designed on regions conserved among the primates with forward and reverse primer positioned at different exons (Table S2). The *Callithrix jacchus* and *Macaca fascicularis* genomes are not yet available. Hence, to acquire transcript sequence information for primer design, we accessed an almost complete *Callithrix jacchus* assembly; The Genome Sequencing Center, Washington University Medical School (http://genome.wustl.edu/pub/organism/Primates/Callithrix_jacchus/assembly/Callithrix_jacchus-2.0.2/). Human exon sequences were used as baits, and Stand-alone BLAST, (NCBI, ftp://ftp.ncbi.nlm.nih.gov/blast/executables/, expectation value (E) set to 10^−6^) was used to extract the marmoset gene sequences. Sequences from the closely related species *Macaca mulatta* available in Ensembl (http://ensembl.org) were used instead of *Macaca fascicularis*. Primers were designed using Primer Express 2.0 (Applied Biosystems).

RNA (1.0 µg) from four males and four females were reversely transcribed to cDNA in a 10 µl reaction volume using Multiscribe RT enzyme (Applied Biosystems) following the manufacturer's instructions. Non-template controls (NTCs) were employed (RT reaction minus Multiscribe RT enzyme) for each individual sample and gene to control for DNA contaminations. RT samples were diluted 1∶20 in nucleace-free water. For relative quantification, a dilution series of cDNA sample was prepared individually for each of the three primate species. Actin-β (ACTB) was used as internal reference. Transcript levels were quantified using ABI Prism 7000 sequence detection system (Applied Biosystems). Reaction conditions: 4.0 µl cDNA sample, 1 x SYBR Green Power Mix (Applied Biosystems), 0.30 µM of each primer, nuclease free water (Invitrogen) up to a total reaction volume of 25 ul. Temperature program: 2 min at 50°C, 10 min at 95°C, 40 repeats of 15 s at 95°C and 60 s at 60°C. ABI Prism 7000 SDS Software was used to extract the qPCR data. The expression level of each selected gene was analyzed with the following linear model,
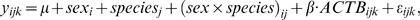
where y_ijk_ is the log transformed mRNA quantity of individual k within sex_i_ and species_j_, ACTB_ijk_ refers to the log transformed mRNA quantity of the references gene ACTB and *ε_ijk_* to the corresponding regression coefficient.

### Analysis of Biological Categories in the Conserved Sexually Dimorphic Genes

To identify overrepresented categories among the genes with conserved sex differences in brain gene expression we used the functional annotation tool on *DAVID* [23]. All genes uploaded to DAVID (n = 85), were annotated by the software. Two sets of reference lists were used: all genes on the microarray and the 50% of genes on the microarray with highest intensities. The following settings were applied: Functional Annotation Chart; thresholds: Count = 2, EASE = 0.05.

### Prediction of Conserved Estrogen and Androgen Cis-Response Elements in the Conserved Sexually Dimorphic Genes

For prediction and analysis of promoter regions in the conserved sexually dimorphic genes we used *Prometheus* which is a flexible system to predict functional cis-regulatory regions in groups of co-regulated genes [25]. The input for the system is a list of genes suspected to be related by a common mechanism, a target and reference species for phylogenetic footprinting and the parameters for the Transcription Factor Binding Site (TFBS) searching. The software searches the orthologous genes for the selected species and retrieves genomic sequences in the two species from Ensembl Database [29]. The sequences are retrieved with 10,000 bases upstream the transcription initiation site and 5,000 downstream the 3′ end. Intronic regions are also included. Next, the sequences are aligned using the LAGAN pairwise aligner [45] and presence of transcription factor binding sites in the conserved regions is predicted by using weight matrices [25]. JASPAR database provides the TF weight matrices [29],[46] used to search in the alignments. In our case, a list of human (*Homo sapiens*) genes was provided (85 genes in Figure 4) and *Macaca mulatta* was the species used for phylogenetic footprinting comparison. To be able to process one gene, the sequence and genomic coordinates for both species (*Homo sapiens* and *Macaca mulatta*) are needed. From the original input list 61 genes were fully annotated in Ensembl Database.

The TFBS searching was done for a set of 129 model matrices using 95% of sequences conservation and 80% threshold for the sequence and matrix model similarity. The supplied results (Table S3) correspond to 10,000 bases upstream the transcription initiation site plus the gene plus 5,000 bases downstream the 3′ end in the 61 annotated genes.

### Data Deposition

Microarray data is publicly available on the EMBL-EBI ArrayExpress database with accession number E-MEXP-1182 and conform to the MIAME guidelines.

## Supporting Information

Figure S1 Evolutionaty relationships among primates. A schematic view of the evolutionary relationships among primates. The species used in our study include; human (*Homo sapiens*, a Great ape), macaque (*Macaca fascicularis*, an Old World monkey), and marmoset (*Callithrix jacchus*, a New World monkey). The names of the groups included in our analysis are circled. The colors of the circles correspond to the colors presented in Figure 1, showing the experimental design. The figure is modification of Figure 34.25, s. 675, Purves et al., Life the Science of Biology, Courier Companies Inc., 7th edition (2004).
(8.05 MB TIF)

Figure S2 MA plots. MA plots for two arrays for each species before and after sub-array intensity dependent normalization of M, showing log-transformed R/G-ratio (M = log2R−log2G) on the y-axis and mean intensity (A = (log2R+log2G)/2 ) on the x-axis. The characters in the upper right corner of each MA plot denote microarray ID numbers.
(4.93 MB TIF)

Figure S3 Analysis of data quality of 24 microarray hybridizations. We calculated the mean background subtracted intensity value from all the spots in each array (mean Ab) and the fraction of missing signals for the arrays (% missing probes). The figure shows that the two arrays that included human female F4 presented 50.2 and 35.6% of missing probes, while the average % of signal loss for all the other 22 arrays was only 6.4%. Intensity values for arrays including F4 were also lower. We therefore removed female 4 from all subsequent analysis. The colors of the points correspond to the colors presented in Figure 1, showing the experimental design.
(2.76 MB TIF)

Table S1 Single species analysis. Genes with sexually dimorphic expression.
(0.63 MB XLS)

Table S2 qPCR primers.
(0.05 MB PDF)

Table S3 Prediction of conserved estrogen and androgen cis- response elements in the conserved sexually dimorphic genes.
(0.11 MB XLS)
