# Supplementary figures and images for: An Evolutionarily Conserved Sexual Signature in the Primate Brain
Source: PLoS Genet. 2008 Jun 20;4(6):e1000100. doi: 10.1371/journal.pgen.1000100 (PMC2413013; doi:10.1371/journal.pgen.1000100)

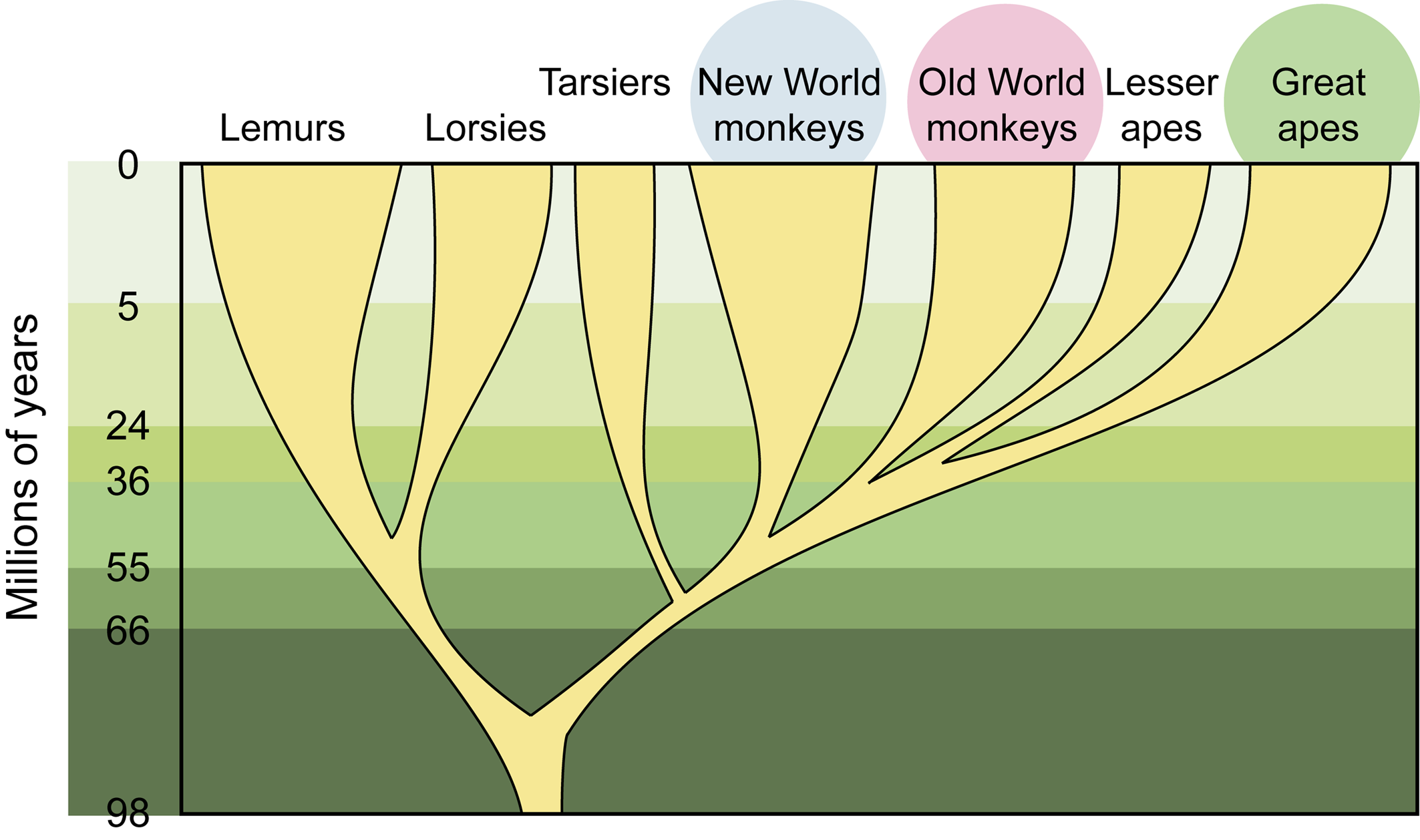

Supplement: Figure S1 — Evolutionaty relationships among primates. A schematic view of the evolutionary relationships among primates. The species used in our study include; human (Homo sapiens, a Great ape), macaque (Macaca fascicularis, an Old World monkey), and marmoset (Callithrix jacchus, a New World monkey). The names of the groups included in our analysis are circled. The colors of the circles correspond to the colors presented in Figure 1, showing the experimental design. The figure is modification of Figure 34.25, s. 675, Purves et al., Life the Science of Biology, Courier Companies Inc., 7th edition (2004). (8.05 MB TIF) [file pgen.1000100.s001.tif]

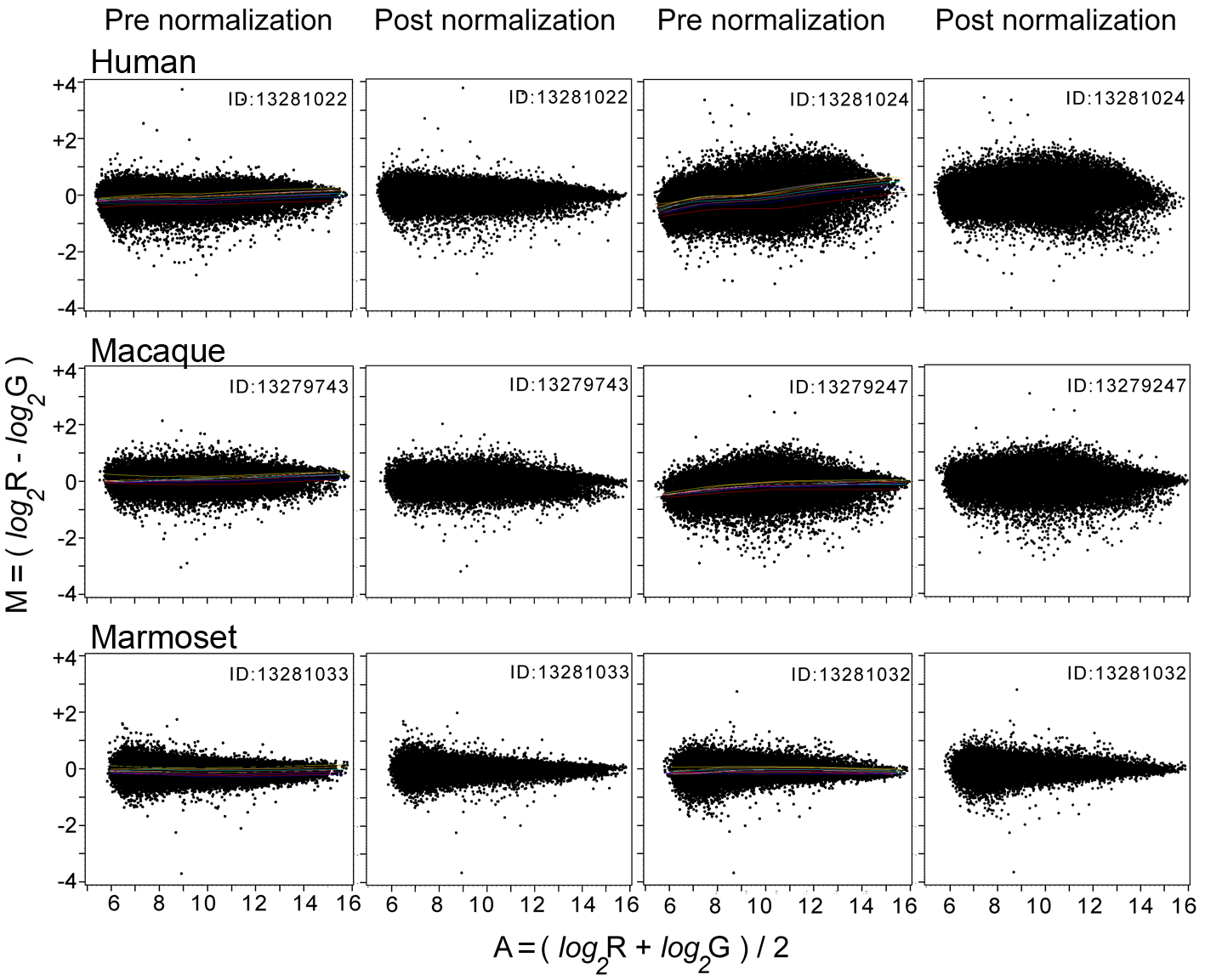

Supplement: Figure S2 — MA plots. MA plots for two arrays for each species before and after sub-array intensity dependent normalization of M, showing log-transformed R/G-ratio (M = log2R−log2G) on the y-axis and mean intensity (A = (log2R+log2G)/2 ) on the x-axis. The characters in the upper right corner of each MA plot denote microarray ID numbers. (4.93 MB TIF) [file pgen.1000100.s002.tif]

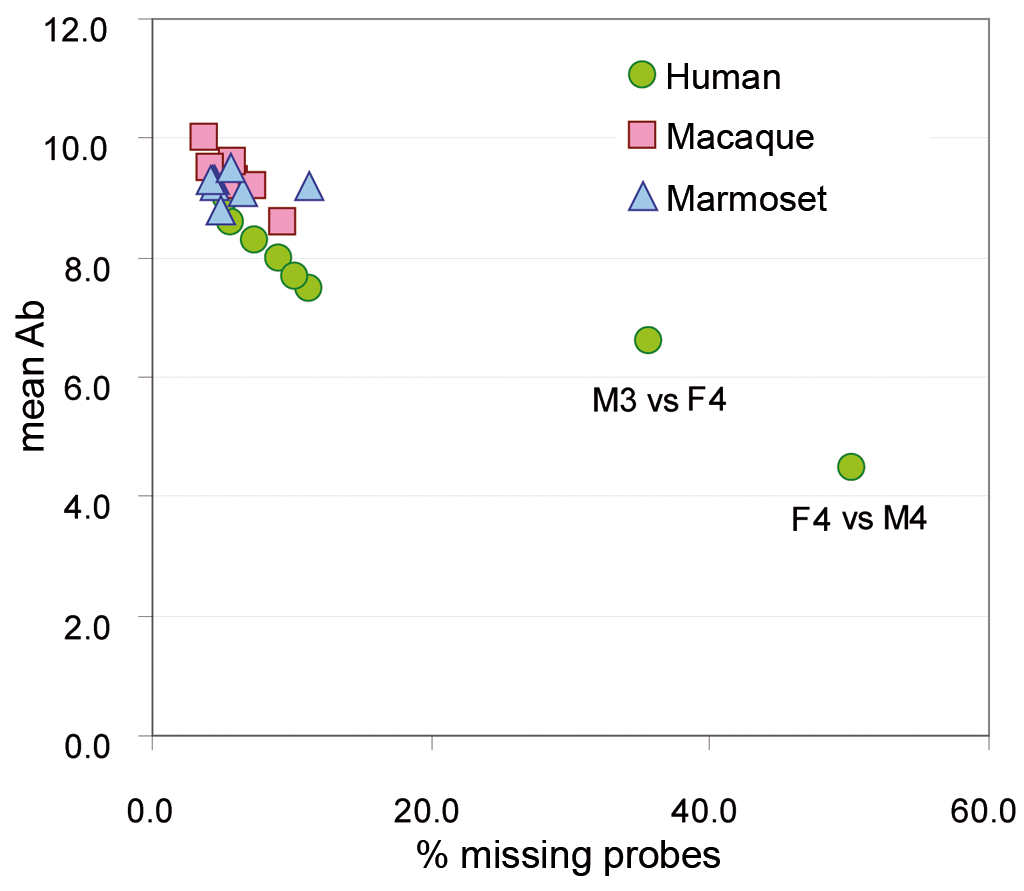

Supplement: Figure S3 — Analysis of data quality of 24 microarray hybridizations. We calculated the mean background subtracted intensity value from all the spots in each array (mean Ab) and the fraction of missing signals for the arrays (% missing probes). The figure shows that the two arrays that included human female F4 presented 50.2 and 35.6% of missing probes, while the average % of signal loss for all the other 22 arrays was only 6.4%. Intensity values for arrays including F4 were also lower. We therefore removed female 4 from all subsequent analysis. The colors of the points correspond to the colors presented in Figure 1, showing the experimental design. (2.76 MB TIF) [file pgen.1000100.s003.tif]
