# Supplementary material for: An Evolutionarily Conserved Sexual Signature in the Primate Brain
Source: PLoS Genet. 2008 Jun 20;4(6):e1000100. doi: 10.1371/journal.pgen.1000100 (PMC2413013; doi:10.1371/journal.pgen.1000100)
Supplement: Table S2 — qPCR primers. (0.05 MB PDF) [file pgen.1000100.s005.pdf]

Primer Name	Sequence (5' to 3')
ATCB Forw.	GGC ATC CAC GAA ACT ACC T
ATCB Rev.	CAA GAA AGG GTG TAA CGC AAC
FGF12 Forw.	TGG TTT CTG GGA CTC AAT AAA GA
FGF12 Rev.	TGA ACG CCC TTG TTT TTC TC
NKIRAS1 Forw.	CAG TTA CAT CTT TAT GAC ACC AGA GG
NKIRAS1 Rev.	CCA CTT CAG CGT CCA CTT G
AOF1 Forw.	GCG TGC TGA TGT CTG TGA TT
AOF1 Rev.	CCC CAC TTC CAC CTG TCT T
SLC6A1 Forw.	CCC CTA TCT CTG CGG GAA AA
SLC6A1 Rev.	CCA CGC CCT TGA ACA TAG GA
EPHX1 Forw.	TCA GAT GAG GAG ATC CAC GA
EPHX1 Rev.	TCC GCC AGT AGG AGA TGA CT
MAP1A Forw.	GCT GCC GCC TCC ATG A
MAP1A Rev.	CCA GGA TGA TGG TCT CTA GGA TCT

Table S2. qPCR primers.
The table contains the primer sequences used in the qPCR experiments.
